# Dietary fiber intake and risks of proximal and distal colon cancers: A meta-analysis

**DOI:** 10.1097/MD.0000000000011678

**Published:** 2018-09-07

**Authors:** Yu Ma, Mingyue Hu, Lingna Zhou, Sunkai Ling, Yuan Li, Bo Kong, Peilin Huang

**Affiliations:** a Department of Oncology, School of Medicine, Southeast University, Nanjing, China; b Department of Surgery, School of Medicine, Technical University Munich (TUM), Munich, Germany.

**Keywords:** dietary fiber, distal colon cancer, meta-analysis, prospective studies, proximal colon cancer

## Abstract

The purpose of this study was to conduct a systematic review and meta-analysis of studies investigating the relationship between dietary fiber intake and subsite-specific colon cancer.

The PubMed database was searched to identify relevant cohort studies published from inception to August 2016 in order to examine individually the association between dietary fiber intake and the risk of proximal colon cancer (PCC), and that between dietary fiber intake and the risk of distal colon cancer (DCC). We searched the reference lists of the studies included in our analysis as well as those listed in the published meta-analyses. A random-effects model was used to compute summary risk estimates. Heterogeneity was assessed using *I*^*2*^ and Q statistics. Publication bias was assessed with the Egger's and Begg's tests, with a *P* value of *P* < .10 indicating publication bias. All statistical tests were 2-sided.

We identified and included 11 prospective cohort studies in the final meta-analysis. The risks of PCC and DCC among individuals in the highest dietary fiber intake quartile/quintile were 14% (relative risk [RR] = 0.86, 95% confidence interval [CI] = 0.78–0.95) and 21% (RR = 0.79, 95% CI = 0.71–0.87) lower, respectively, than those among individuals with the lowest dietary fiber intake. In a subgroup analysis, the inverse association observed in the sex-based subgroup was apparent only for men with PCC. Dietary fiber intake was inversely associated with DCC for both men and women. In addition, dietary fiber intake appeared to be inversely associated with PCC only in European countries, whereas this association was observed for DCC in both European countries and the United States.

Our findings reveal that dietary fiber intake is associated inversely with the risk of both PCC and DCCs.

## 1 Introduction

Globally, colorectal cancer has the third highest incidence among all types of cancers, with about 1.2 million patients diagnosed in 2008 alone and comprised approximately one-tenth of all diagnosed cancers in 2017.^[1]^ Furthermore, only alcohol and red meat consumption have been shown to increase the risk of colorectal cancer, according to previous epidemiological studies.^[2]^

The association between dietary fiber intake and colorectal cancer has been intensively investigated. Increased fiber intake may lead to a dilution of fecal carcinogens, reduced transit time, and increased bacterial fermentation of fiber to short-chain fatty acids with anticarcinogenic properties.^[3,4]^ A 2011 meta-analysis indicated a 10% reduction in the risk of colorectal cancer for each 10 g/day intake of total dietary fiber and cereal fiber, and an approximately 20% reduction for a daily increment of 3 servings (90 g/day) of whole grain, with further reductions for higher intake.^[5]^ Because cancers of the proximal and distal colon have been proposed to be 2 distinct cancer types with different genetic and environmental risk factors,^[6]^ it is important to define whether dietary fiber intake is differentially associated with the risk of developing each of these colon cancer types. However, to date, the majority of studies that investigated a possible link between dietary fiber intake and the risk of proximal colon cancer (PCC) and distal colon cancer (DCC) have yielded inconclusive results.^[7–17]^ Therefore, to clarify these differential associations, we conducted a systematic review and meta-analysis.

## 2 Materials and methods

### 2.1 Data sources and searches

We performed a literature search using the following key words for articles in the PubMed database from its inception through August 2016 (www.ncbi.nlm.nih.gov/pubmed): (”dietary fiber” or ”dietary fiber”), (”risk” or ”incidence”), (”colon cancer” or ”colorectal cancer”), and (”cohort study” or ”prospective study”). No language restrictions were imposed. In addition, we searched the reference lists of the studies that were included in our analysis as well as those listed in the published meta-analyses. The statistical review of the study was performed by Dr Xinhua Qu from Shanghai Ninth People's Hospital, Shanghai Jiao Tong University School of Medicine. Ethical approval and patient consent were not required, as this study was done on published data.

### 2.2 Study selection

To be included in this analysis, a study must have fulfilled the following criteria: original article; cohort study; classification of colon cancer into no more than 2 types (i.e., PCC and DCC); inclusion of data for relative risk (RR); and the corresponding 95% confidence interval (CI). Two authors independently assessed articles to determine whether the study met the eligibility criteria outlined above. Differences were resolved by discussion.

### 2.3 Data extraction

The following data were extracted from each included eligible study: first author's last name, publication year, study location, study name, study period, sex and age of participants, dietary assessment, total number of colon cancers, numbers of PCCs and DCCs, range of exposure, RRs or hazard ratios of dietary fiber, and corresponding 95% CIs for the association of dietary fiber intake with the risks of PCC and DCC, and confounders adjusted (Adj) for in the analysis.

### 2.4 Statistical analysis

We used random-effects meta-analyses to calculate the summary RRs for the associations between dietary fiber and the risks of PCC and DCC.^[18]^ Men and women were treated separately as 2 samples, and risk estimates for both sexes were included in the primary analysis.^[12]^ We extracted the maximally Adj RR with corresponding 95% CI for the highest versus lowest quartile/quintile of dietary fiber intake for use in the primary analyses. Heterogeneity was assessed using *I*^*2*^ and Q statistics.^[19]^*I*^*2*^ values represent the amount of total variation explained by variation among studies, with a value of >50% indicating severe heterogeneity, and a value of <25% indicating the absence of significant heterogeneity. Publication bias was assessed with the Egger's and Begg's tests, with *P* < .10 indicating publication bias.^[20]^

### 2.5 Subgroup analyses

Four prespecified subgroup analyses were conducted using the following criteria to define the subgroups: sex; duration of follow-up (<10 years or >10 years); study location (the United States or European countries); the numbers of PCC and DCC cases (<400 or >400); and confounders used for adjustment, including age, body mass index (BMI), smoking, exercise, education level, calcium, folate, red meat intake, and hormone replacement therapy (in women only).

All statistical tests were 2-sided, and a *P* value less than .05 was considered significant. All analyses were performed with STATA version 12.0 (Stata Corp, College Station, TX).

## 3 Results

### 3.1 Study selection

**Figure 1:**
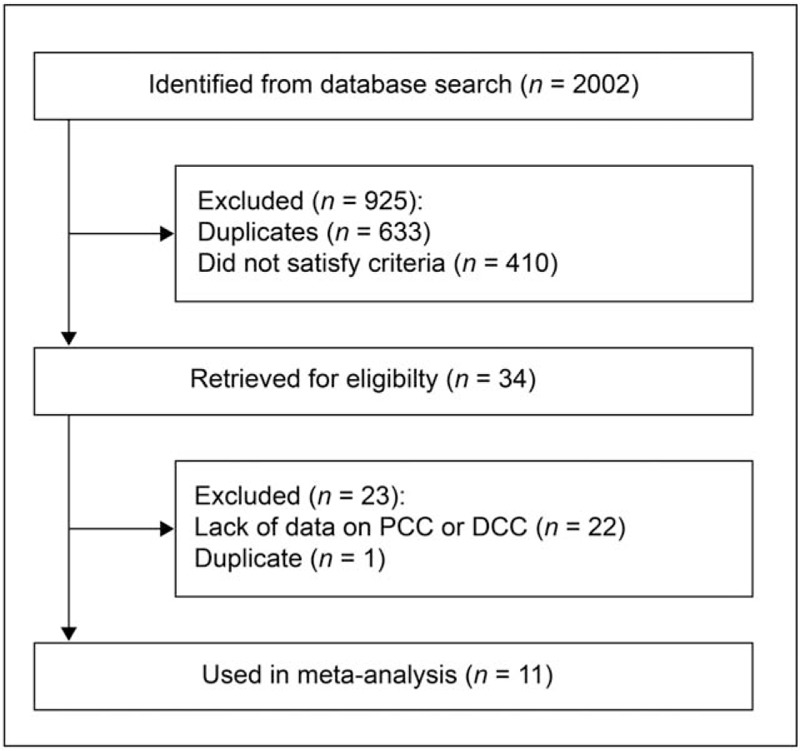
Study selection flowchart. DCC = distal colon cancer, PCC = proximal colon cancer.

A total of 2002 articles were identified in the literature search (Fig. 1). Of these, 925 were excluded for various reasons, and 410 did not satisfy the inclusion criteria. After eliminating duplicate studies, 34 unique articles remained. After reviewing the full text of these articles, we excluded 23 studies that were not relevant to this review and another 22 studies that lacked sufficient data on PCC and DCC. The 11 remaining studies were included in the primary meta-analysis.^[7–17]^

### 3.2 Study characteristics

The main characteristics of the 11 studies included in the primary meta-analysis are displayed in Table 1 . Seven studies were conducted in the United States^[7,8,11–14,17]^; 4 studies were conducted in Europe (1 in 10 European countries,^[16]^ 2 in Sweden,^[9,10]^ and 1 in Denmark^[15]^); 6 studies involved only women,^[7–11,14]^ and 5 involved both men and women,^[12,13,15–17]^ 1 of which reported sex-specific results.^[12]^

**Table 1:**
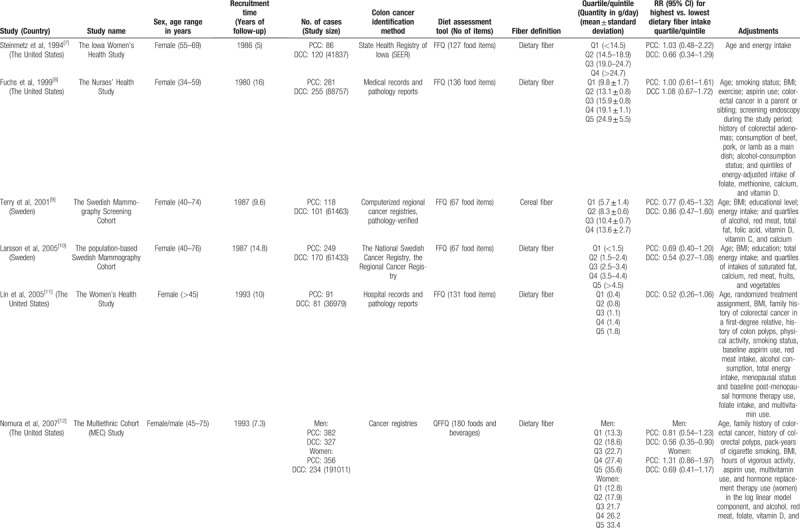
Characteristics of studies examining fiber intake the risk of proximal and distal colon cancer.

**Table 1 (Continued):**
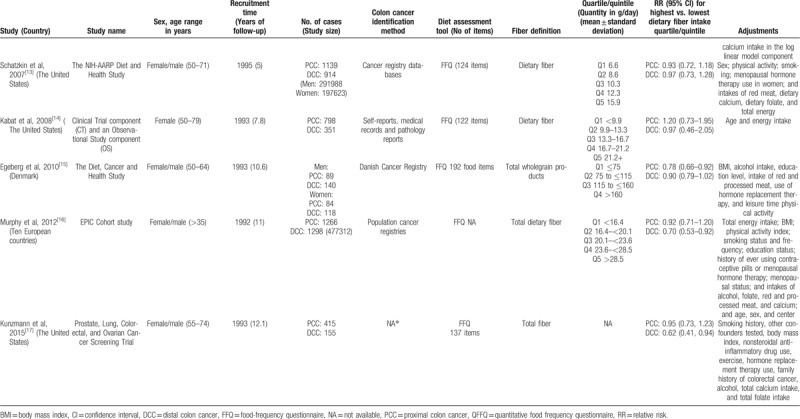
Characteristics of studies examining fiber intake the risk of proximal and distal colon cancer.

### 3.3 Meta-analyses

#### 3.3.1 Primary meta-analysis

**Figure 2:**
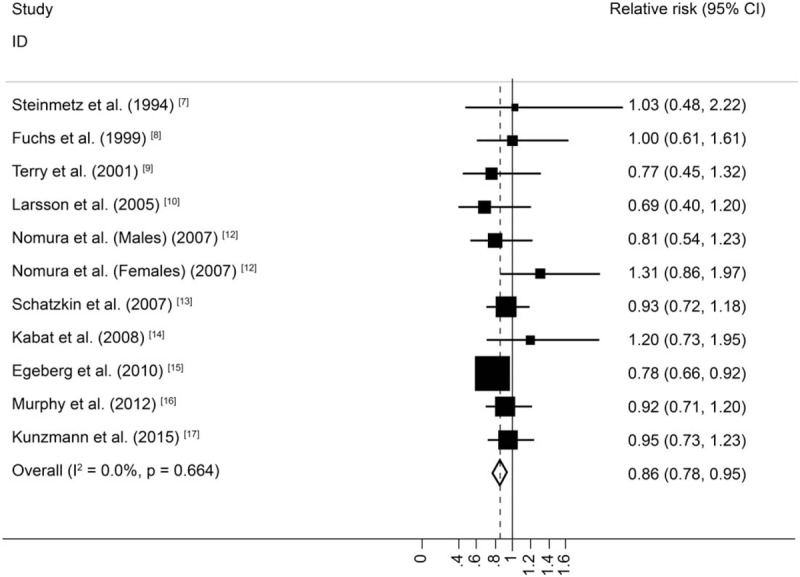
Random-effects meta-analysis of prospective cohort studies that examined dietary fiber intake and proximal colon cancer risk.

**Figure 3:**
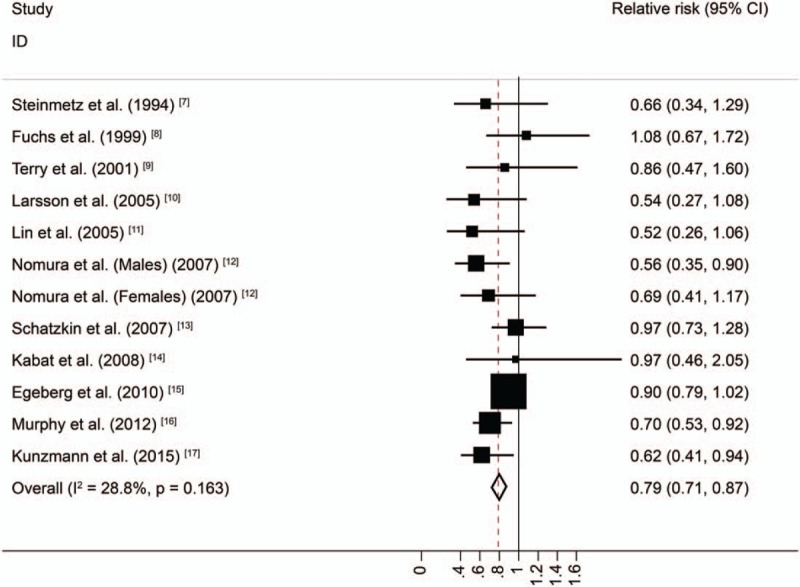
Random-effects meta-analysis of prospective cohort studies that examined dietary fiber intake and distal colon cancer risk.

The summary RR of PCC for individuals with the highest dietary fiber intake relative to those with the lowest intake was 0.86 (95% CI = 0.78–0.95), with no heterogeneity (*I*^*2*^ = 0%; *P* = .664; Fig. 2). The pooled odds ratios of DCC indicated that the risk of DCC among individuals with the highest dietary fiber intake was 21% lower than that among individuals with the lowest dietary fiber intake (RR = 0.79, 95%CI = 0.71–0.87), again with low heterogeneity (*I*^*2*^ = 28.8%; *P* = .163; Fig. 3).

### 3.4 Publication bias

In this meta-analysis, evidence of publication bias was detected. The Egger's test for the various studies examining DCC and PCC, yielded *P* values of.064 and.230, respectively. In addition, for studies examining DCC and PCC, Begg's test yielded *P* values of .75 and .00, respectively.

### 3.5 Subgroup meta-analyses

In subgroup analyses by sex, duration of follow-up, study location, the numbers of PCCs and DCCs, and confounders, dietary fiber intake was inversely associated with the risk of colorectal cancer in most subgroups, with no evidence of significant heterogeneity between subgroups in meta-regression analyses. The inverse association observed in the sex-based subgroup was apparent only for men with PCC. Dietary fiber intake was inversely associated with DCC for both men and women. In addition, dietary fiber intake appeared to be inversely associated with PCC only in European countries, whereas this association was observed for DCC in both European countries and the United States. In the subgroups of studies that Adj for age, BMI, and smoking, however, dietary fiber intake was inversely associated with DCC, with significant heterogeneity between subgroups (*P* = .044, .014 and.045 respectively; Table 2).

**Table 2:**
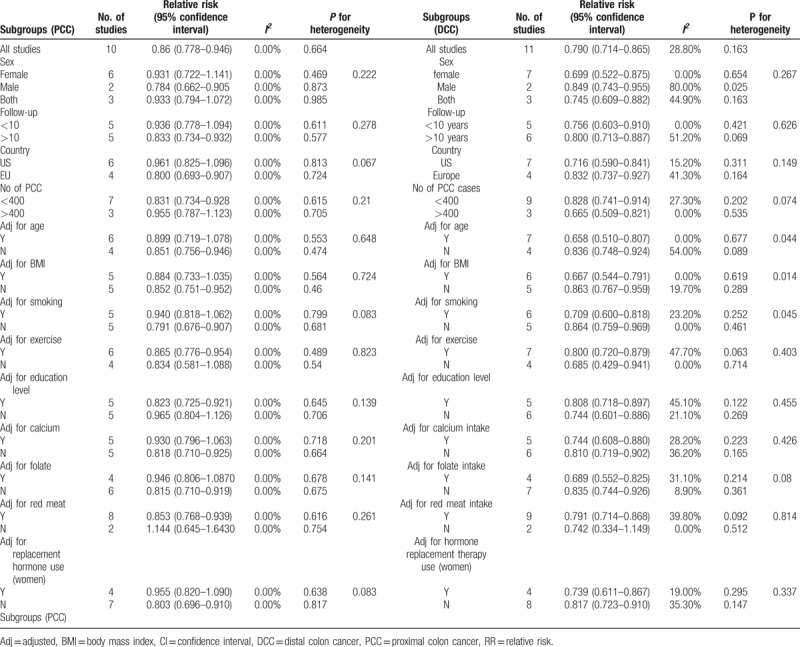
Subgroup analyses of fiber intake and risk of PCC and DCC.

## 4 Discussion

According to the findings of this meta-analysis and systematic review, anatomical subsite has no apparent association between the incidence of colon cancer and dietary fiber intake. Based on the summary risk estimates of the 11 analyzed studies, individuals with the smallest intake of dietary fiber had a 21% and 14% higher risk of DCC and PCC, respectively, compared to those with maximal dietary fiber intake. Previous studies have suggested that dietary fiber has stronger anticancer effects on PCC for European males, while little difference has been found in its anticancer effects on DCC for males and females.

Despite finding a similar effect of dietary fiber on the incidence of both DCC and PCC in this study, this was contradictory to the results of the examined studies within in this meta-analysis. However, there were some inconsistencies identified in the results of these previous studies: some results suggested that the anticancer effect of dietary fiber on DCC was stronger compared with its effect on PCC, while other results suggested either the opposite trend or no significant difference at all. Due to the limitation in the total number of studied cases, there has been deficient statistical significance in colon subsite's positive results.^[11]^ The precise reason for dietary fiber's stronger anticancer effect on PCC for male patients remains unclear. Some investigators have suggested that one reason for this explanation could be that there are typically fewer females diagnosed with left-sided colon cancer, ^[12]^ while others have suggested that estrogen replacement therapy may have protective effects against colon cancer.^[21,22]^ In addition, differences between genders in the composition and synthesis of bile acid may explain the disparity in colon cancer incidence between males and females.^[23]^ Further, the precise reason for differing results regarding dietary fiber with DCC and PCC may be elucidated by subgroup analysis in various study locations. Based on the results of this analysis, the above association was consistent; however, a sufficient amount of location-specific cases have not been examined. Given that various environmental risk factors exist for colon cancer, the interaction between these factors and colon cancer risk may be influenced by dietary fiber intake.^[12]^

Some biological mechanisms may explain the differences in the association of various factors and DCC and PCC. DCC and PCC are embryologically, morphologically, physiologically, and biochemically different.^[6]^ According to some studies, fast-degrading and fast-fermenting soluble fiber polysaccharides can lead to highly concentrated short-chain fatty acids in the proximal colon. However, more highly concentrated short-chain fatty acids produced by dietary fiber with slow degradation have also been detected in the distal colon. Individuals with a lower fecal pH tend to have a lower incidence of colon cancer.^[24]^ Moreover, insoluble non-starch polysaccharides, which are the most basic elements that comprise dietary fiber, are able to transfer resistant starch to distal colonic regions for fermentation, thus lowering ammonia concentration and increasing the concentration in these areas, where tumors are most likely to occur.

There were some limitations in this study. First, our primary meta-analysis could have been more statistically heterogeneous. However, according to the results of our subgroup analysis that Adj for smoking history, BMI, and age, dietary fiber intake was negatively correlated to the incidence of DCC, which showed high heterogeneity. Second, the quintile/quartile ranges of dietary fiber intake varied considerably from study to study, and this could have added heterogeneity to the pooled analysis. Third, food-frequency questionnaires (FFQs) were adopted in all the included studies, and when considering their susceptibility to measurement errors, the information collected with questionnaires could have impacted the association of dietary fiber intake with colon cancer risk. Fourth, our meta-analysis did not include many studies examining dietary fiber association with the incidence of colon cancer due to a lack of investigating colon cancer subsite. Despite excluding these studies, we obtained nearly identical results with another recent meta-analysis concerning dietary fiber's association with the incidence of colon cancer, in which separate results for DCC and PCC were not reported.^[5]^ Under this circumstance, the studies contained in our meta-analysis are representative of other meta-analyses. A previous meta-analysis that included 6 studies reported a 0.88 summary risk estimate for colorectal cancers, compared with our respective summary RRs of 0.79 and 0.86 for PCC and DCC, respectively.

Regardless of study limitations mentioned above, our analysis presents some convincing findings. Each study included in our meta-analysis came utilized a prospective design, which successfully prevented the occurrence of recall and selection biases. The majority of these studies involved the evaluation of multiple critical confounders, including the intake of red and processed meat, smoking habits, and BMI. Moreover, most of these studies involved a large number of colon cancer patients, as well as a relatively long follow-up period, which strengthened the statistical power of our study.

In conclusion, the results of this systematic review and meta-analysis present strong and consistent evidence that dietary fiber is associated with reduced risks of both proximal colon and distal colon cancers, and that the association between dietary fiber intake and risk of colon cancer does not differ by cancer subsite.

## Acknowledgments

We would like to thank Editage (www.editage.com) for English language editing and Publication Support. The statistical review of the study was performed by Dr Xinhua Qu from Shanghai Ninth People's Hospital, Shanghai Jiao Tong University School of Medicine.

## Author contributions

**Conceptualization:** Yu Ma, Mingyue Hu, Lingna Zhou.

**Formal analysis:** Yu Ma, Mingyue Hu, Peilin Huang.

**Investigation:** Yu Ma, Yuan Li.

**Resources:** Bo Kong.

**Writing – original draft:** Yu Ma, Lingna Zhou, Sunkai Ling.
